# High-mobility ZrInO thin-film transistor prepared by an all-DC-sputtering method at room temperature

**DOI:** 10.1038/srep25000

**Published:** 2016-04-27

**Authors:** Peng Xiao, Ting Dong, Linfeng Lan, Zhenguo Lin, Wei Song, Dongxiang Luo, Miao Xu, Junbiao Peng

**Affiliations:** 1 State Key Laboratory of Luminescent Materials and Devices (South China University of Technology), Wushan Road 381#, Tianhe District, Guangzhou, China

## Abstract

Thin-film transistors (TFTs) with zirconium-doped indium oxide (ZrInO) semiconductor were successfully fabricated by an all-DC-sputtering method at room temperature. The ZrInO TFT without any intentionally annealing steps exhibited a high saturation mobility of 25.1 cm^2^V^−1^s^−1^. The threshold voltage shift was only 0.35 V for the ZrInO TFT under positive gate bias stress for 1 hour. Detailed studies showed that the room-temperature ZrInO thin film was in the amorphous state with low carrier density because of the strong bonding strength of Zr-O. The room-temperature process is attractive for its compatibility with almost all kinds of the flexible substrates, and the DC sputtering process is good for the production efficiency improvement and the fabrication cost reduction.

There have been growing interests in transparent oxide semiconductors (TOSs) for their potential application in thin-film transistors (TFTs) backplanes of flat-panel displays (FPDs) such as active matrix organic light-emitting diodes (AMOLEDs)^1^,^2^,^3^ and liquid-crystal displays (LCDs)^4^,^5^. InGaZnO (IGZO) is one of the most common TOSs owing to its advantages of relatively high mobility compared to amorphous silicon, good uniformity, visible-light transparency, and low cost^6^,^7^,^8^,^9^,^10^. However, the processing temperature of IGZO TFTs is still too high for most of the transparent plastic substrates, such as polyethersulphone (PES), polyethylene naphthalate (PEN), polyethylene terephthalate (PET), and polycarbonate (PC), which require processing temperature below 180 °C^11^,^12^,^13^. On the other hand, the mobility of IGZO (usually around 10 cm^2^V^−1^s^−1^) is still not enough for the future high-resolution, high-frame rate, or 3D displays which require TFT backplanes with mobility of higher than 20 cm^2^V^−1^s^−1^.

To address the above issues, many groups have attempted to attain high-mobility and low-temperature TFTs using InO_x_-based TOSs, such as In_2_O_3_^14^,^15^,^16^, InZnO^17^,^18^, Sn-InZnO^19^, Hf-InZnO^20^,^21^, Si-InZnO^22^,^23^, Zr-InZnO^24^,^25^, InWO^26^ and InSiO^27^,^28^, as the channel materials. However, the stability of most of the low-temperature TOS-TFTs are not good enough for applications in FPDs. In another aspect, most of the TOS films are fabricated with RF sputtering which is not favorite in the mass-production lines because of the low deposition rate and the RF-radiation dangers.

In this paper, TFTs with zirconium-doped indium oxide (ZrInO) channel layer were prepared by an all-DC-sputtering method at room temperature. Instead of RF sputtering, DC sputtering was chosen to deposit ZrInO films for its advantages of high deposition rate, good reproducibility, good uniformity for multi-component film, no need of power matching, and no RF-radiation danger^29^,^30^,^31^. The gate dielectric layer of the ZrInO TFTs was prepared by room-temperature anodization, rather than by plasma-enhanced chemical vapor deposition (PECVD) which requires another expensive vacuum instrument and would cause environment pollution due to the requirement of expensive, toxic, flammable, and explosive gases.

The Zr element of ZrInO is considered to be a superior oxygen binder to suppress the formation of oxygen vacancies for its low electronegativity (1.4)^24^ and strong bonding strength with oxygen (the bonding energy of Zr-O is as high as 776 KJ/mol)^32^. In comparison, the electronegativity of In is 1.78^33^, and the bonding strength of In-O is only 348 KJ/mol^28^. In addition, the radius of Zr^4+^ is 0.72 Å which is close to that of In^3+^ (0.79 Å), so incorporation of Zr into In_2_O_3_ will not cause serious lattice distortion.

## Results and Discussion

**Figure 1:**
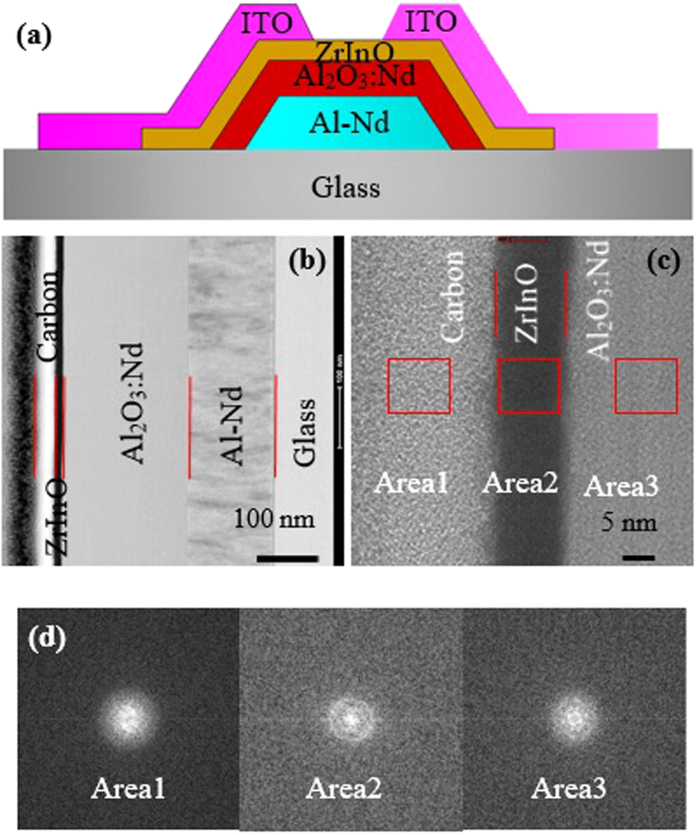
(**a**) Schematic structure of ZrInO-TFT with anodic gate dielectric, (**b**) Cross-sectional STEM image of ZrInO-TFT, (**c**) HR-TEM image of ZrInO/Al_2_O_3_:Nd cross-sectional structure, (**d**) FFT patterns obtained from Area 1–3.

Figure 1(a) shows the schematic cross-sectional structure of the TFTs with ZrInO channel layer, and Fig. 1(b,c) show the scanning transmission electron microscopy (STEM) and high-resolution transmission electron microscopy (HR-TEM) images of the ZrInO/Al_2_O_3_:Nd/Al-Nd cross sectional structure, respectively. Both of the STEM and HR-TEM images revealed an uniform and continuous ZrInO/Al_2_O_3_:Nd interface without pinholes or hillocks. Figure 1(d) shows the fast Fourier transform (FFT) patterns for different areas indicated in Fig. 1(c), revealing the presence of nanocrystalline ZrInO and Al_2_O_3_:Nd domains.

**Figure 2:**
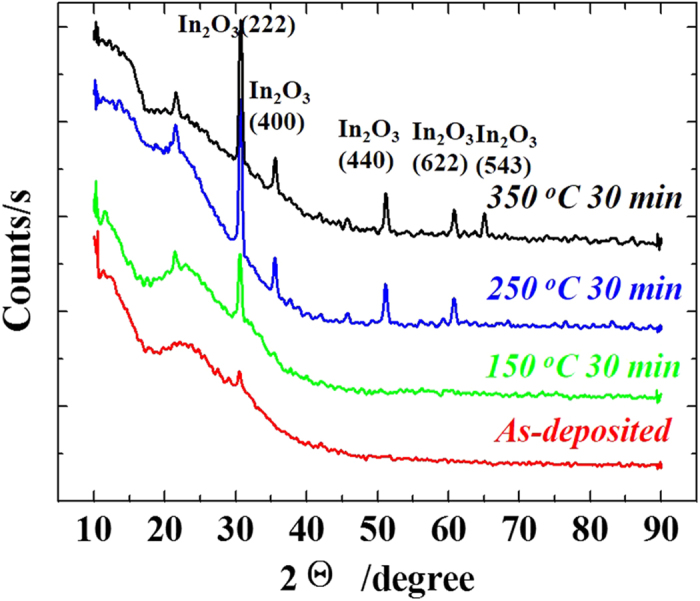
XRD patterns of ZrInO films deposited on glass substrate, with different annealing temperatures.

Figure 2 shows the X-ray diffraction (XRD) patterns of the ZrInO thin films (140 nm) with different annealing temperature. The as-deposited ZrInO thin film was almost in amorphous phase. The ZrInO thin film annealed at 150 °C exhibited a clear crystalline peak at 30.71^o^, which coincided well with the (222) peaks of In_2_O_3_ bixbyite structure. It implied that almost no lattice distortion took place after incorporation of Zr into In_2_O_3_, which was ascribed to the similar ionic radii of Zr^4+^ (0.72 Å) and In^3+^ (0.79 Å). The 250 °C-annealed thin film exhibited more peaks at 35.47^o^, 51.04^o^, and 60.68^o^, corresponding to the (400), (440), and (622) peaks of In_2_O_3_ bixbyite structure, respectively. As the annealing temperature increased to 350 °C, another peak for In_2_O_3_ (543) was found. The results suggest that the film structure of ZrInO is almost the same as that of In_2_O_3_, and increasing the annealing temperature will cause crystallization which is not good for the uniformity of the electrical performances.

Field-emission scanning electron microscopy (FESEM) was used to study the surface morphology of ZrInO thin films with different annealing temperature, as shown in Figure S1. All the ZrInO thin films showed uniform and dense surfaces, which is critical to obtain high performance TFTs. In addition, the changes of the grain size with the annealing temperature were consistent with the result of XRD and TEM experiments above.

**Figure 3:**
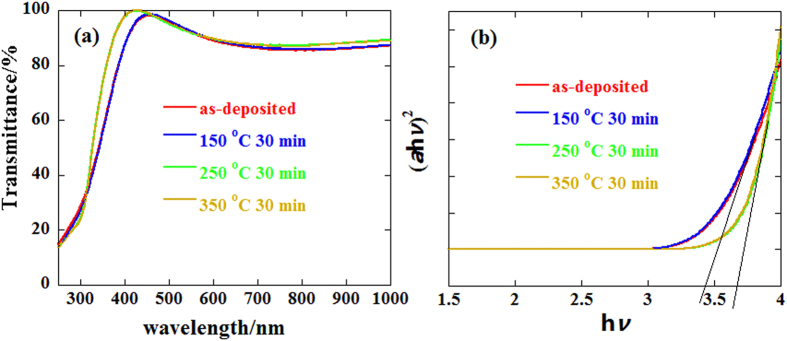
(**a**) Transmittance spectra and (**b**) band gap of ZrInO thin films with respect to various annealing temperature.

Figure 3(a) shows the transmittance spectra of the 140 nm-thick ZrInO thin films coated on a quartz substrate with different annealing temperature. All ZrInO thin films were optically transparent and showed an average transmittance of exceeding 85% at wavelengths ranging between 400 and 1000 nm, indicating that ZrInO thin film could be used as an active channel layer for fully transparent displays. The optical band gap (*E*_*g*_) of the ZrInO thin films were estimated from the Tauc Plot (Equation 1)^34^,


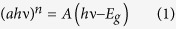


where A is the constant of material; *α* is the absorption coefficient; h is Planck’s constant and *ν* is the frequency; and n is the coefficient value (herein, n = 2). Figure 3(b) shows the curves of (*α*h*ν*)^2^ versus h*ν*. The as-deposited and 150 °C-annealed ZrInO thin films showed the same *E*_*g*_ of 3.42 eV. As the annealing temperature increased to 250 °C, the values of *E*_*g*_ increased sharply to 3.67 eV which was the same as that of the 150 °C-annealed one. The variation of *E*_*g*_ was ascribed to the higher degree of crystallinity when the annealing temperature reached 250 °C, as discussed above.

**Figure 4:**
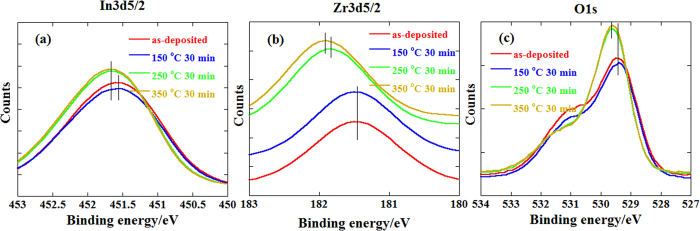
XPS spectra for (**a**) In 3d_5/2_, (**b**) Zr 3d_5/2_ and (**c**) O 1 s for ZrInO samples before and after annealed at 150 °C, 250 °C and 350 °C for 30 min.

X-ray photoelectron spectroscopy (XPS) analysis was also conducted to observe the relationship between the oxygen bonding states and the annealing temperature. Figure 4(a–c) show In 3d_5/2_, Zr 3d_5/2_, and O 1 s spectra of ZrInO thin films with different annealing temperature, respectively. Compared to the as-deposited and 150 °C annealed ZrInO thin films, the 250 °C and 350 °C annealed thin films showed a smaller hump at 531 ± 0.1 eV which is corresponding to the oxygen vacancy (V_o_). And the content of V_o_ of ZrInO thin films with different annealing temperature were obtained from Figure S2 and listed in Table 1. Compared to the as-deposited ZrInO thin film with V_o_ content of 38.3%, the V_o_ content of 150 °C-annealed one slightly increased up to 38.7%. And the V_o_-content sharply decreased down to 29.1% and 19% for 250 °C and 350 °C annealed ones, respectively. The same phenomena were observed in the spin-orbit split XPS data of In 3d_5/2_ and Zr 3d_5/2_, as shown in Fig. 4(a,b), respectively. Both of the peaks of In 3d_5/2_ and Zr 3d_5/2_ in ZrInO thin film shifted toward the high binding energy direction as the annealing temperature increased, indicating an increase of the coordination number of the Zr or In with the increasing annealing temperature. It should be noted that the binding energy shift (Δ*E*_*b*_) of In 3d_5/2_ was only 0.11 eV, much smaller than that of Zr 3d_5/2_ (0.46 eV), implying that the Zr ions would gain more oxygen ions compared to indium ions.

**Figure 5:**
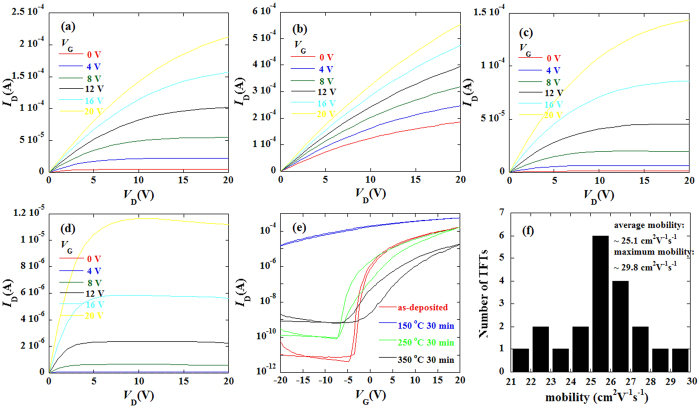
The output curves of the as-deposited (**a**), 150 °C-annealed (**b**), 250 °C-annealed (**c**), 350 °C-annealed (**d**) ZrInO TFTs; (**e**) the transfer characteristics of ZrInO TFTs with different annealing temperature; (**f**) the mobility distribution for 20 ZrInO TFTs.

Figure 5(a–d) shows the output characteristics (*V*_GS_ = 0–20 V in steps of 4 V) obtained from ZrInO TFTs with different annealing temperature. The drain current (*I*_D_) in the output curves increased linearly in the low drain voltage (*V*_DS_) regime without obvious current-crowding phenomenon, which indicated ohmic contacts were formed between ZrInO channel and ITO electrodes. Figure 5(e) shows the corresponding transfer characteristics of the ZrInO TFTs with different annealing temperature. The detailed properties of the ZrInO TFTs were summarized in Table 2. The as-deposited ZrInO TFT exhibited good performance with an average saturation mobility (*μ*_sat_) of 25.1 cm^2^V^−1^s^−1^ (from 20 devices, as shown in Fig. 5(f)), a threshold voltage (*V*_th_) of −0.94 V, a sub threshold swing (*SS*) of 0.42 V/decade, and an on/off current ratio (*I*_*on*_*/I*_*off*_) of 3.3 × 10^7^. The small hysteresis in transfer characteristic between forward and reverse sweeps was ascribed to few adsorption state^35^ (such as O^2−^ and O^−^ etc.) on the ZrInO back channel because of its insensitivity to air. As the annealing temperature increased to 150 °C, the ZrInO TFT became conductive, which indicated that the carrier concentration was too high to be depleted. Further increased the annealing temperature to 250 or 350 °C, the turn-on voltage shifted towards the positive direction, but hysteresis and the off current (*I*_*off*_) increased largely. These results suggest that the room-temperature processed ZrInO TFTs have the best performance which is attributed to the lowest carrier density. It is worth noting that the undoped In_2_O_3_ TFTs exhibited high conductivity (unable to be turn off) even without any annealing steps. Therefore, the Zr dopant has an effect of suppressing free carrier generation.

**Figure 6:**
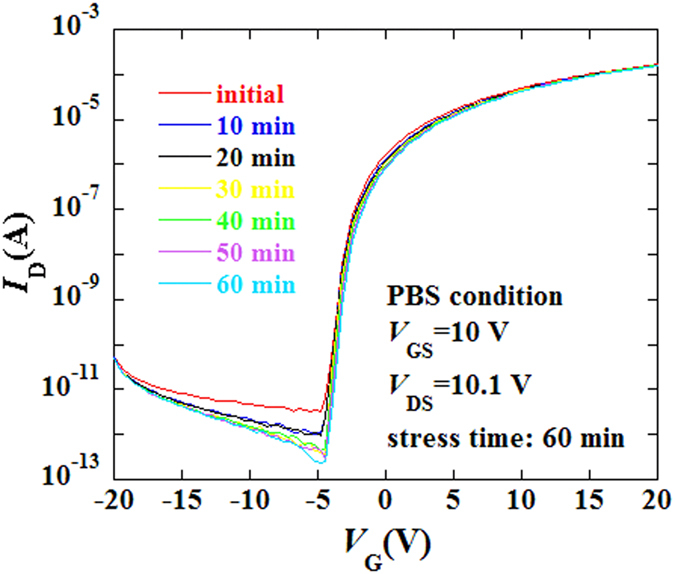
Variations in the transfer characteristics of ZrInO TFT under PBS test. *V*_GS_ = 10 V and *V*_DS_ = 10.1 V for a duration of 60 min.

Figure 6 shows the electrical stability of the unannealing ZrInO TFT under positive bias stress (PBS). During the test, a positive bias (*V*_GS_ = 10 V, *V*_DS_ = 10.1 V) was applied as an electrical stress for 60 min, and the transfer curves were recorded every 10 min. It is clearly to see that ZrInO TFT without passivation layer exhibited excellent electrical stability with a threshold voltage shift of only 0.35 V, indicating that the ZrInO material was insensitive to air (H_2_O or O_2_). It suggests that the room-temperature, all DC-sputtered ZrInO TFTs is stable enough for the backplanes of AMOLEDs.

In conclusion, ZrInO thin film prepared by DC magnetron sputtering were investigated as an active channel layer for TFTs. The structural properties of ZrInO thin films were analyzed using XRD, SEM and TEM, which showed an increase in the crystallinity as the annealing temperature increased. The average transmittance of the ZrInO thin films were over 85% in the wavelength ranging between 400 and 1000 nm. The as-deposited ZrInO TFTs exhibited an average *μ*_*sat*_ of 25.1 cm^2^V^−1^s^−1^; a *V*_*th*_ of −0.94 V; a *SS* of 0.42 V per decade and an *I*_*on*_*/I*_*off*_ of 3.3 × 10^7^ with excellent positive bias stress stability (Δ*V*_*th*_ = 0.35 V/h). The room-temperature processes without any intentionally annealing steps show a great potential for the applications in the flexible displays, and the DC sputtering method is good for the production efficiency improvement and cost reduction.

## Methods

ZrO_2_ and In_2_O_3_ powders were weighed in a stoichiometric ratio (ZrO_2_:In_2_O_3_ = 1:99 wt.%) and were thoroughly mixed after griding. Then the powders were pressed into a pellet and were sintered at ~1450 °C for 48 h to form a ZrInO target. Then the ZrInO target was fixed in a sputter. The ZrInO semiconducting thin film was prepared by DC magnetron sputtering with a power of 35 W (0.1 A, 350 V), a gas mixture ratio of Ar:O_2_ of 8:0.5 sccm, and a working pressure of 0.45 Pa.

TFTs with ZrInO channel layers were fabricated with bottom-gate structure, as shown in Fig. 1(a). A layer of 300-nm-thick Al-Nd alloy was deposited on a glass substrate by DC magnetron sputtering and patterned by wet etch. Then, a layer of Nd:Al_2_O_3_ was prepared by an anodization process as the gate dieletric layer^36^,^37^. After that, a 20-nm-thick ZrInO active layer was deposited onto Nd:Al_2_O_3_ by DC magnetron sputtering and patterned by shadow mask. For the source/drain electrodes, a 380-nm-thick ITO thin film was sputtered through a shadow mask defining a channel width/length (*W/L*) of 300/300 μm. The whole preparation process was performed at room temperature without intentionally annealing.

The surface morphology and structure properties of the oxide thin films were characterized by scanning electron microscopy (SEM, Hitachi S-4800) and transmission electron microscopy (TEM, FEI Titan Themis 200) equipped with an energy dispersive X-ray spectrometer (EDS), respectively. The crystal structures were confirmed via X-ray diffraction (XRD, Philips X pert pro M). The optical properties of the ZrInO thin films were analyzed using ultraviolet fluorescence spectrometer (Shimadzu, UV-3600). X-ray photoelectron spectroscopy (XPS, Thermo Scientific, Escalab 250 XI) using monochromatic Al Ka radiation (~1486.6 eV) was used to examine chemical composition of the ZrInO thin films. The XPS data were calibrated with C 1 s peak at ~284.6 eV. The electrical measurements were performed using a semiconductor parameter analyzer (Agilent 4155 C) in air.

## Additional Information

**How to cite this article**: Xiao, P. *et al.* High-mobility ZrInO thin-film transistor prepared by an all-DC-sputtering method at room temperature. *Sci. Rep.* **6**, 25000; doi: 10.1038/srep25000 (2016).

## Supplementary Material

Supplementary Information

## Figures and Tables

**Table 1:** Summary of the peak positions, the peak shifts (Δ*E*_b_) for the Zr, In and O element, and the oxygen vacancy (V_o_) content in ZrInO thin films with different annealing temperature obtained via XPS measurements.

Post-annealed temperature	In 3d_5/2_ (eV)	Δ*E*_b_ (In) (eV)	Zr 3d_5/2_ (eV)	Δ*E*_b_ (Zr) (eV)	O 1 s (eV)	Δ*E*_b_ (O) (eV)	V_o_-content (%)
As-deposited	451.56	0	181.46	0	529.43	0	38.3
150 °C	451.56	0	181.46	0	529.43	0	38.7
250 °C	451.67	0.11	181.83	0.37	529.64	0.21	29.1
350 °C	451.67	0.11	181.92	0.46	529.64	0.21	19.0

**Table 2:** Electrical characteristics of ZrInO TFTs with different annealing temperature.

Post-annealed temperature	*V*_on_ (V)	*V*_th_ (V)	*I*_*on*_*/I*_*off*_	*μ*_sat_(cm^2^V^−1^s^−1^)	SS (V decade^−1^)	Δ*V*_th_ (V)
As-deposited	−4.67	−0.94	3.3 × 10^7^	25.1	0.42	0.35
150 °C	N/A	N/A	N/A	15.5	N/A	N/A
250 °C	−7.33	1.52	1.7 × 10^6^	25.1	0.61	N/A
350 °C	−6.00	3.98	2.6 × 10^4^	4.4	2.28	N/A

## References

[b1] Liao C. *et al.* Mirrored OLED pixel circuit for threshold voltage and mobility compensation with IGZO TFTs. Microeletron. J. 46, 923−927 (2015).

[b2] Lin C.-L. *et al.* New a-IGZO Pixel Circuit Composed of Three Transistors and One Capacitor for Use in High-Speed-Scan AMOLED Displays. J. Display Technol. 11, 1031−1034 (2015).

[b3] Genoe J. *et al.* Integarated Line Driver for Digital Pulas-Width Modulation Driven AMOLED Displays on Flex. IEEE J. Solid-State Circuits. 50, 282−290 (2015).

[b4] Kim S. C. *et al.* Short channel amporphous In-Ga-Zn-O thin-film transistor arrays for ultra-high definition active matrix liquid crystal displays: Electrical properties and stability. Solid-State Electron. 111, 67–75 (2015).

[b5] Shishido H. *et al.* High aperture ratio LCD display using In-Ga-Zn-Oxide TFTs without storage capacitor. Proc. SID Dig. 1128−1131 (2010).

[b6] Lan L. & Peng J. High-Performance Indium–Gallium–Zinc Oxide Thin-Film Transistors Based on Anodic Aluminum Oxide. IEEE Trans. Electron Devices. 58, 1452−1455 (2011).

[b7] Kim M. *et al.* High mobility bottom gate InGaZnO thin film transistors with SiO_*x*_ etch stopper. Appl. Phys. Lett. 90, 212114 (2007).

[b8] Nomura K. *et al.* Room-temperature fabrication of transparent flexible thin-film transistors using amorphous oxide semiconductors. Nature. 432, 488−492 (2004). 15565150 10.1038/nature03090

[b9] Kamiya T. *et al.* Present status of amorphous In-Ga-Zn-O thin-film transistors. Sci. Technol. Adv. Mater. 11, 044305 (2010). 10.1088/1468-6996/11/4/044305 PMC5090337 27877346

[b10] Kwon J.-Y. Lee D.-J. & Kim K.-B. Transparent amorphous oxide semiconductor thin film transistor. Electron. Mater. Lett. 7, 1–11 (2011).

[b11] Choi M. C. Kim Y. & Ha C. S. Polymers for flexible displays: From material selection to device applications. Prog. Polym. Sci. 33, 581−630 (2008).

[b12] Choi M. C. *et al.* New colorless substrates based on polynorbornene-chlorinated polyimide copolymers and their application for flexible displays. J. Poym. Sci. part A: Polym. Chem. 48, 1806−1814 (2010).

[b13] Jin H. S., Chang J. H. & Kim J. C. Synthesis and characterization of colorless polyimide nanocomposite films containing pendant trifluoromethyl groups. Macromol. Res. 16, 503−509 (2008).

[b14] Weiher R. L. Electrical Properties of Single Crystals of Indium Oxide. J. Appl. Phys. 139, 504 (1954).

[b15] Dhananjay & Chu C.-W. Realization of In_2_O_3_ thin film transistors through reactive evaporation process. Appl. Phys. Lett. 91, 132111 (2007).

[b16] Nayak P. K. *et al.* High performance In_2_O_3_ thin film transistors using chemically derived aluminum oxide dielectric. Appl. Phys. Lett. 103, 033518 (2013).

[b17] Barquinha P. *et al.* Effect of UV and visible light radiation on the electrical performances of transparent TFTs based on amorphous indium zinc oxide. J. Non-Cryst. Solids. 352, 1756−1760.

[b18] Paine D. C. *et al.* Amorphous IZO-based transparent thin film transistors. Thin Solid Films. 516, 5894−5898 (2008).

[b19] Oh S. *et al.* Anomalous behavior of negative bias illumination stress instability in an indium zinc oxide transistor: A cation combinatorial approach. Appl. Phys. Lett. 101, 092107 (2012).

[b20] Kim C.-J. *et al.* Amorphous hafnium−indium−zinc oxide semiconductor thin film transistors. Appl. Phys. Lett. 95, 252103 (2009).

[b21] Chong E. *et al.* High stability of amorphous hafnium−indium−zinc−oxide thin film transistor. Appl. Phys. Lett. 96, 152102 (2010).

[b22] Chong E. Chun Y. S. & Lee S. Y. Amorphous silicon−indium−zinc oxide semiconductor thin film transistors processed below 150 °C. Appl. Phys. Lett. 97, 102102 (2010).

[b23] Chong E., Kim S. H. & Lee S. Y. Role of silicon in silicon−indium−zinc−oxide thin−film transistor. Appl. Phys. Lett. 97, 252112 (2010).

[b24] Park J.-S. *et al.* Novel ZrInZnO Thin-film Transistor with Excellent Stability. Adv. Mater. 21, 329−333 (2009).

[b25] Jeong T. H. *et al.* Study of the Effects of Zr-Incorporated InZnO Thin-Film Transistors Using a Solution Process. Jpn. J. Appl. Phys. 50, 070202 (2011).

[b26] Aiakwa S. *et al.* Thin-film transistors fabricated by low-temperature process based on Ga- and Zn- free amorphous oxide semiconductor. Appl. Phys. Lett. 102, 102101 (2013).

[b27] Aikawa S. *et al.* Effects of dopants in InO_x_-based amorphous oxide semiconductors for thin-film transistor applications. Appl. Phys. Lett. 103, 172105 (2013).

[b28] Mitoma N. *et al.* Stable amorphous In_2_O_3_-based thin-film transistors by incorporating SiO_2_ to suppress oxygen vacancies. Appl. Phys. Lett. 104, 102103 (2014).

[b29] Moon Y.-K. *et al.* Application of DC Magnetron Sputtering to Deposition of InGaZnO Films for Thin Film Transistor Devices. Jpn. J. Appl. Phys. 48, 031301 (2009).

[b30] Aoi T. *et al.* DC sputter deposition of amorphous indium−gallium−zinc−oxide (a-IGZO) films with H_2_O introduction. Thin Solid Films. 518, 3004−3007 (2010).

[b31] Yasuno S. *et al.* Physical Properties of Amorphous In−Ga−Zn−O Films Deposited at Different Sputtering Pressures. Jpn. J. Appl. Phys. 52, 03BA01 (2013).

[b32] Parthiban S. & Kwon J.-Y. Amorphous boron−indium−zinc−oxide active channel layers for thin-film transistor fabrication. J. Mater. Chem. C. 3, 1661 (2015).

[b33] Lin Z. *et al.* Studies on Nd_x_In_1-x_O_3_ semiconducting thin films prepared by rf magnetron sputtering. Appl. Phys. Lett. 105, 142104 (2014).

[b34] Tauc J. Optical properties and electronic structure of amorphous Ge and Si. Mater. Res. Bull. 3, 37−46 (1968).

[b35] Jeong J. *et al.* Origin of threshold voltage instability in indium-gallium-zinc oxide thin film transistors. Appl. Phys. Lett. 93, 123508 (2008).

[b36] Luo D. *et al.* Role of Rare Earth Ions in Anodic Gate Dielectrics for Indium-Zinc-Oxide Thin-Film Transistors. J. Electrochem. Soc. 159, H502 (2012).

[b37] Lan L. *et al.* Low-Voltage High-Stability Indium-Zinc Oxide Thin-Film Transistor Gated by Anodized Neodymium-Doped Aluminum. IEEE Electron Device Lett. 33, 827–829 (2012).

