# Supplementary material for: High-mobility ZrInO thin-film transistor prepared by an all-DC-sputtering method at room temperature
Source: Sci Rep. 2016 Apr 27;6:25000. doi: 10.1038/srep25000 (PMC4846996; doi:10.1038/srep25000)
Supplement: Supplementary Information [file srep25000-s1.pdf]

Supplementary Information

High-mobility ZrInO thin-film transistor prepared by an all-DC-sputtering method at room temperature

Peng Xiao [1], Ting Dong[1], Linfeng Lan[1,*], Zhenguo Lin[1], Wei Song[1], Dongxiang Luo[1], Miao Xu[1] & Junbiao Peng[1,*]

[1]State Key Laboratory of Luminescent Materials and Devices (South China University of Technology), Wushan Road 381#, Tianhe District, Guangzhou, China.

Correspondence and requests for materials should be addressed to L.L. (lanlinfeng@scut.edu.cn) or J.P. (psjbpeng@scut.edu.cn)

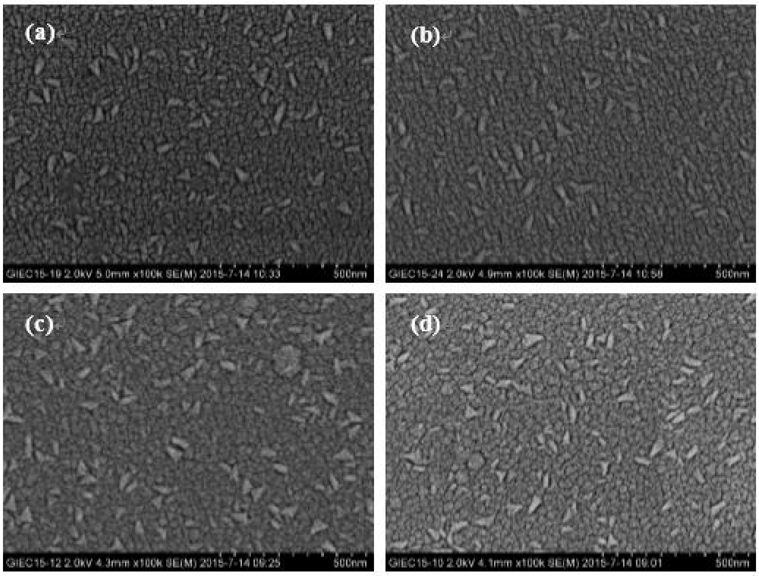

Figure S1. Field emission electron microscopy (FESEM) pictures of 140 nm-thick-ZrInO thin films with different annealing temperature: (a) as-deposited; (b) 150 °C, 30 min; (c) 250 °C, 30 min; (d) 350 °C, 30 min.

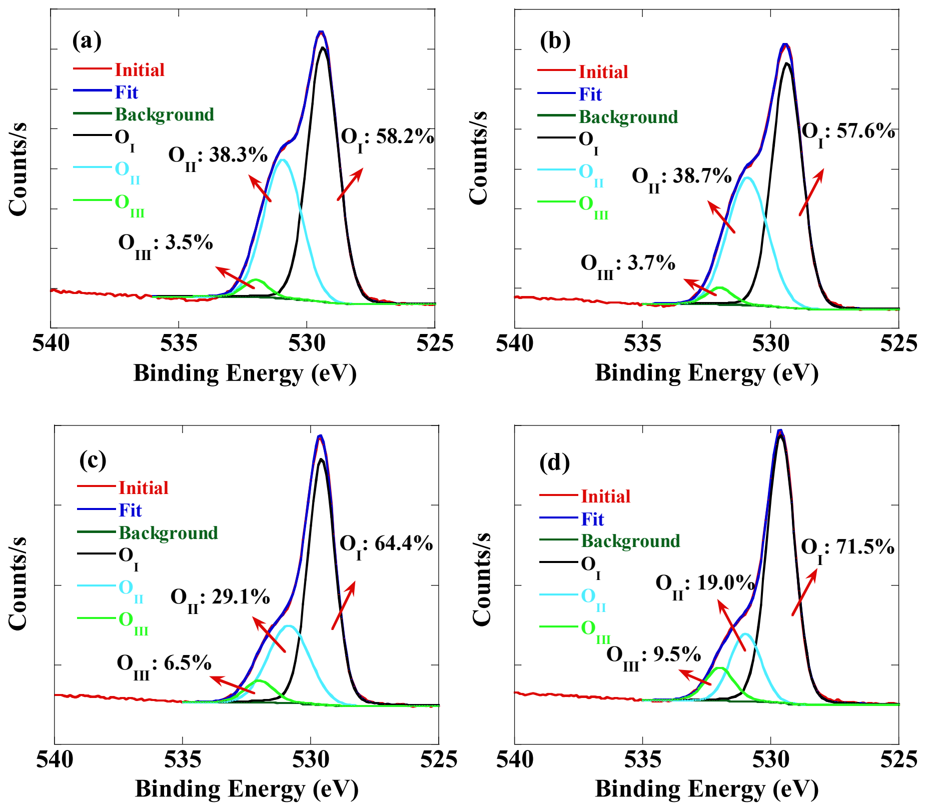

Figure S2. The XPS spectra of the O1s core levels for ZrInO samples (a) before and after annealed at (b) 150 ºC, (c) 250 ºC and (d) 350 ºC for 30min; O_I: the oxygen in stoichiometry of the ZrInO films; O_{II}: oxygen vacancies. O_{III}: OH groups attached to ZrInO surface.
